# Development of an efficient conjugation-based genetic manipulation system for *Pseudoalteromonas*

**DOI:** 10.1186/s12934-015-0194-8

**Published:** 2015-01-23

**Authors:** Pengxia Wang, Zichao Yu, Baiyuan Li, Xingsheng Cai, Zhenshun Zeng, Xiulan Chen, Xiaoxue Wang

**Affiliations:** Key Laboratory of Tropical Marine Bio-resources and Ecology, Guangdong Key Laboratory of Marine Materia Medica, RNAM Center for Marine Microbiology, the South China Sea Institute of Oceanology, Chinese Academy of Sciences, Guangzhou, 510301 China; State Key Laboratory of Microbial Technology, Marine Biotechnology Research Center, Shandong University, Jinan, 250100 China; University of Chinese Academy of Sciences, Beijing, 100049 China

**Keywords:** *Pseudoalteromonas*, Conjugation, Genetic manipulation, Marine bacteria

## Abstract

*Pseudoalteromonas* is commonly found throughout the world’s oceans, and has gained increased attention due to the ecological and biological significance. Although over fifty *Pseudoalteromonas* genomes have been sequenced with an aim to explore the adaptive strategies in different habitats, *in vivo* studies are hampered by the lack of effective genetic manipulation systems for most strains in this genus*.* Here, nine *Pseudoalteromonas* strains isolated from different habitats were selected and used as representative strains to develop a universal genetic manipulation system. Erythromycin and chloramphenicol resistance were chosen as selection markers based on antibiotics resistance test of the nine strains. A conjugation protocol based on the RP4 conjugative machinery in *E. coli* WM3064 was developed to overcome current limitations of genetic manipulation in *Pseudoalteromonas*. Two mobilizable gene expression shuttle vectors (pWD2-oriT and pWD2Ery-oriT) were constructed, and conjugation efficiency of pWD2-oriT from *E. coli* to the nine *Pseudoalteromonas* strains ranged from 10^−6^ to 10^−3^ transconjugants per recipient cells. Two suicide vectors, pK18*mobsacB*-Cm and pK18*mobsacB*-Ery (with *sacB* for counter-selection), were constructed for gene knockout. To verify the feasibility of this system, we selected gene or operon that may lead to phenotypic change once disrupted as targets to facilitate *in vivo* functional confirmation. Successful deletions of two genes related to prodigiosin biosynthesis (*pigMK*) in *P. rubra* DSM 6842, one biofilm related gene (*bsmA*) in *P.* sp. SM9913, one gene related to melanin hyperproduction (*hmgA*) in *P. lipolytica* SCSIO 04301 and two flagella-related genes (*fliF* and *fliG*) in *P.* sp. SCSIO 11900 were verified, respectively. In addition, complementation of *hmgA* using shuttle vector pWD2-oriT was rescued the phenotype caused by deletion of chromosomal copy of *hmgA* in *P. lipolytica* SCSIO 04301. Taken together, we demonstrate that the vectors and the conjugative protocol developed here have potential to use in various *Pseudoalteromonas* strains.

**Electronic supplementary material:**

The online version of this article (doi:10.1186/s12934-015-0194-8) contains supplementary material, which is available to authorized users.

## Background

Genus *Pseudoalteromonas* belongs to the *Gammaproteobacteria* class with thirty-eight recognized species reported so far [1,2]. *Pseudoalteromonas* is ubiquitous in the marine environment [2-10], and many strains have been isolated from deep sea [8], polar sea [2,9,10], or other extreme marine habitats, highlighting their important and diverse role in marine ecosystems. *Pseudoalteromonas* strains also produce a range of bioactive compounds with antimicrobial, antifouling, or algicidal activities that have attracted global attentions from microbiologist, ecologists and chemists [11]. To date, over fifty *Pseudoalteromonas* genomes have been sequenced, laying a solid foundation for comparative studies on their adaptability to ecological niches as well as for the discovery of novel natural products. Several studies have used ectopic expressing genes in *E. coli* as a complementary means to interrogate genes and their functions in *Pseudoalteromonas* [12,13]. However, the lack of an efficient and universal genetic manipulation system has limited the comparative studies of *Pseudoalteromonas* at the molecular level *in vivo*.

Shuttle vector pWD2 has been successfully isolated previously and can be used as an expression vector in its original derived strain *P.* sp. SM20429 [9]. Direct transfer of non-mobilizable pWD2 to other *Pseudoalteromonas* strains is constrained by the need for electroporation. Electroporation does not seem to work in majority of *Pseudoalteromonas* strains whose growth are usually salt-dependent*.* Based on our current knowledge, to date, gene deletion systems have only been described for two *Pseudoalteromonas* strains, *P. haloplanktis* TAC125 and *P.* sp. SM9913 [14,15]. Both protocols were designed for the construction of strain-specific isogenic knockouts, thus developing a widely applicable genetic manipulation system for *Pseudoalteromonas* now becomes a priority.

A few common features of *Pseudoalteromonas* make genetic manipulation difficult. Harboring multidrug resistance genes and multiple drug efflux pumps in the genome [8,16] can equip cells to survive antibiotic pressure and also can develop further mutations in genes encoding the target sites of antibiotics [17]. Abundant distribution of restriction-modification systems also reduces transformation efficiency by degrading foreign DNAs [18]. In addition, commonly used conjugation protocol does not offer a condition that allow decent growth of the non-marine originated mesophilic donor strain and the marine recipient *Pseudoalteromonas* strains. Solving these problems is critical for developing efficient genetic manipulation systems for *Pseudoalteromonas*.

Bacterial conjugation is a genetic exchange mechanism that requires direct contact between donor and recipient cells. Bacterial conjugation machinery is composed of an *oriT* sequence and *tra* genes [19]. The *oriT* sequence needs to be provided by the plasmid in *cis*, while the *tra* genes, which encode a relaxase, a mating pair formation complex, and a type IV coupling protein, can be provided in *cis* or in *trans*. The relaxase cleaves the *nic* site within the *oriT* sequence and covalently attaches to the 5′ end of the transferred strand to produce a single-strand DNA (ssDNA)-relaxase complex with other auxiliary proteins; this is termed the relaxosome [19]. The type IV coupling protein mediates the connection between the relaxasome and the mating pair formation complex, the latter being the secretion system that transfers ssDNA-relaxase complex into recipient cells [20]. Since the DNA transferred by conjugation is single-stranded instead of double-stranded transferred by electroporation, thus it could reduce the possible degradation by restriction-modification systems which preferably degrading double-stranded DNAs [18]. As a result, conjugation techniques have been widely used for genetic manipulations in Gram-negative bacteria and have also been reported in several Gram-positive bacteria (reviewed in [21]).

Here, we present an efficient conjugation-based genetic manipulation system for *Pseudoalteromonas*. Nine *Pseudoalteromonas* strains from different habitats were selected to represent strains from deep-sea sediment, Arctic sea ice, deep-sea hydrothermal vent, Mediterranean coastal water, Antarctic surface seawater, and sediment or surface water in the South China Sea. Based on antibiotic sensitivity test, two different resistance genes are used for selection to construct new vectors for gene expression and gene knockout. A conjugal transfer system with a modified medium using these vectors is developed, and feasibility of this transferring system is confirmed in nine *Pseudoalteromonas* strains. We further demonstrate that targeted deletion mutants are successfully constructed in four *Pseudoalteromonas* strains using this system to facilitate studies of these genes or operons *in vivo,* including *P. rubra* DSM 6842, *P.* sp. SM9913, *P. lipolytica* SCSIO 04301 and *P.* sp. SCSIO 11900. In addition, gene complementation using this system is also confirmed in one deletion mutant of *P. lipolytica* SCSIO 04301.

## Results and discussion

### Antibiotic resistance in different *Pseudoalteromonas* strains

To develop a universal genetic manipulation system for a variety of *Pseudoalteromonas* strains, nine *Pseudoalteromonas* strains were selected as representative strains (Table 1). The ability to resist different antibiotics of each strain was first analyzed in order to find the most suitable resistance markers for maintaining the vectors in proper host (Additional file 1: Table S1). All nine *Pseudoalteromonas* strains were sensitive to 25 μg/ml erythromycin and 30 μg/ml chloramphenicol. None of the nine strains was sensitive to gentamicin, kanamycin, spectinomycin, and tetracycline. Resistance to apramycin and ampicillin is partial in some strains thus not suitable for constructing universal vectors. Taken together, erythromycin and chloramphenicol resistance genes serve as good candidates of constructing vectors for universal gene expression and gene knockout in *Pseudoalteromonas*.

**Table 1:** **Bacterial strains and plasmids used in this study**

**Strains/plasmids**	**Description** ^**a**^	**Reference**
***Pseudoalteromonas*** **strains**		
SM9913	*P.* sp. SM9913, deep-sea sediment at a water depth of 1855 meters near the Okinawa Trough, 20°C	[8]
A37-1-2	*P. arctica* A37-1-2, Arctic sea ice strain, 20°C	[7]
DSM 16099	*P. spiralis* DSM 16099, deep ocean hydrothermal vents of the Juan de Fuca Ridge, 30°C	[35]
DSM 16098	*P. telluritireducens* DSM 16098, deep ocean hydrothermal vents of the Juan de Fuca Ridge, 30°C	[35]
DSM 6842	*P. rubra* DSM 6842, Mediterranean coastal waters off Nice, 25°C	[26,36]
SM20429	*P.* sp. SM20429, plasmid curing mutant of *P.* sp. BSi20429, Arctic sea ice strain, 20°C	[9]
TAC125	*P. haloplanktis* TAC125, Antarctic surface seawater, 20°C	[37]
SCSIO 04301	*P. lipolytica* SCSIO 04301, sediment at 63 m deep in the South China Sea (18°0=N, 109°42=E), 25°C	[28]
SCSIO 11900	*P.* sp. SCSIO 11900, surface mucus layer of the coral at 4 m deep in the South China Sea (18°13=N, 109°28=E) , 25°C	[28]
Δ*bsmA*	*bsmA* gene deletion mutant of *P.*sp. SM9913	This study
Δ*pigM-K*	Deletion mutant of the DNA region containing *pigM*-*K* genes related to prodigiosin biosynthesis in *P. rubra* DSM 6842	This study
Δ*hmgA*	*hmgA* gene deletion mutant of *P. lipolytica* SCSIO 04301	This study
Δ*fliFG*	Deletion mutant of the DNA region containing *fliFG* genes encoding flagellar motor proteins in *P.* sp. SCSIO 11900	This study
***Escherichia. coli*** **strain**		
WM3064	RP4 (tra) in chromosome, DAP^-^, 37°C	[23]
**Plasmids**		
pWD2	*E. coli* and *Pseudoalteromonas* shuttle vector, Amp^r^, Cm^r^	[9]
pHT304	*E. coli* and *Bacillus thuringiensis* shuttle vector, Ampr, Eryr *thuringiensis* shuttle vector, Amp^r^, Ery^r^	[30]
pBBR1MCS-2	Broad-host-range cloning vector, Kan^r^	[22]
pWD2-oriT	pWD2 containing 1.5 kb *Bam*HI fragment with mobilization region from pBBR1MCS-2, Amp^r^, Cm^r^	This study
pWD2Ery-oriT	pWD2-oriT containing a 900bp erythromycin resistant gene replaced the chloramphenicol resistant gene, Amp^r^, Ery^r^	This study
pK18*mobsacB*	Widely used gene knockout vector, Kan^r^	[24]
pK18*mobsacB*-Cm	pK18*mobsacB* containing the chloramphenicol resistant gene from pWD2, Kan^r^, Cm^r^	This study
pK18*mobsacB*-Ery	pK18*mobsacB* containing the erythromycin resistant gene from pHT304, Kan^r^, Ery^r^	This study
pK18Cm-*bsmA*	pK18*mobsacB*-Cm containing the homologous arms of *bsmA* gene of SM9913, Kan^r^, Cm^r^	This study
pK18Ery-*pigM-K*	pK18*mobsacB*-Ery containing the homologous arms of the *pigM*-*pigK* DNA region of DSM 6842, Kan^r^, Ery^r^	This study
pK18Ery-*hmgA*	pK18*mobsacB*-Ery containing the homologous arms of *hmgA* gene of SCSIO 04301, Kan^r^, Ery^r^	This study
pK18Ery-*fliFG*	pK18*mobsacB*-Ery containing the homologous arms of the *fliF*-*fliG* DNA region of SCSIO 11900, Kan^r^, Ery^r^	This study

^a^Amp^r^, ampicillin resistance; Ery^r^, erythromycin resistance; Kan^r^, kanamycin resistance; Cm^r^, chloramphenicol resistance.

Temperature indicates the optimal growth temperature of each strain.

### Construction of mobilizable shuttle vectors for *Pseudoalteromonas*

**Figure 1:**
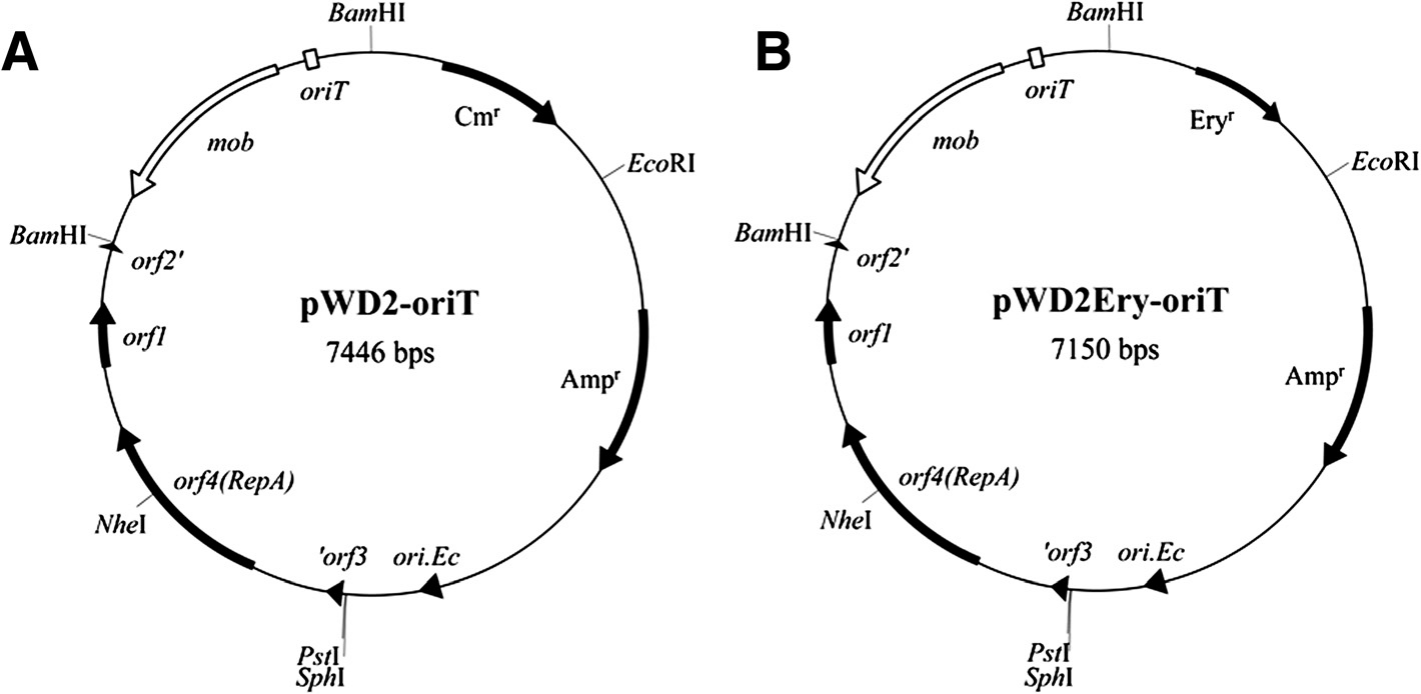
**Maps of the pWD2-oriT (A) and pWD2Ery-oriT (B) mobilizable shuttle vectors.**

Initially we made many attempts to electroplate vector pWD2 [9] to SM9913 and A37-1-2. Transformation efficiencies remained extremely low, despite that electroporation of pWD2 into its original host strain was successful using a similar, previously described method [9]. As noted above, electroporation is of only very limited use in *Pseudoalteromonas*, warranting the use of a conjugationapproach. To conduct conjugation, the pWD2-oriT mobilizable plasmid (Figure 1A) was constructed by inserting the mobilization module from pBBR1MCS-2 [22] into the *E. coli*-*Pseudoalteromonas* shuttle vector pWD2 [9]. The mobilizable module, which contains the *mob* gene and *oriT* sequence, was amplified using the *Bam*HI site-flanking primer set oriT-F/oriT-R (Additional file 1: Table S2) and ligated into the *Bam*HI sites of pWD2. Vector pWD2-oriT therefore acts as a shuttle vector that can be mobilized by the RP4 conjugative machinery in *trans* between *E. coli* and *Pseudoalteromonas.* To offer an alternative selection marker, the pWD2Ery-oriT vector (Figure 1B) was also constructed by replacing the chloramphenicol resistance gene with the erythromycin resistance gene at the same site.

### Conjugation of pWD2-oriT between *E. coli* and *Pseudoalteromonas*

The widely used conjugation donor strain *E. coli* WM3064 has RP4 Tra function integrated in the chromosome, and is an auxotrophic strain whose growth relies on the supplementation of diaminopimelic acid (DAP) in the medium [23]. First, the pWD2-oriT shuttle vector was transformed into *E. coli* WM3064 to obtain *E. coli* WM3064/pWD2-oriT. Using it as a donor strain, pWD2-oriT was then transferred into nine recipient *Pseudoalteromonas* strains individually by intergeneric conjugation. The conjugation protocol was optimized to allow decent simultaneous growth of the donor and recipient strains. Different conjugation temperatures (15, 25, 30, or 37°C) and media with different salt concentrations (LB+DAP, modified LB+DAP, or 2216E+DAP) were tested, respectively. Since *E. coli* WM3064 is able to grow at temperatures as low as 15°C, the optimal mating temperature was thus determined by the optimal growth temperature of the recipient strain (shown in Table 1). Growth of *E. coli* WM3064 and *Pseudoalteromonas* was also affected by the salt concentration in the medium. *E. coli* WM3064 grew very slowly in 2216E+DAP medium which has high salt concentration, and most *Pseudoalteromonas* strains had poor growth in LB medium (Additional file 1: Figure S1). Since viability of the donor strain during mating appeared to be a more important determining factor for conjugation efficiency, modified LB with moderate salt concentration was chosen for *E. coli* WM3064-*Pseudoalteromonas* mating to guarantee growth of both donor and recipient strains (Additional file 1: Figure S1).

To evaluate the conjugation protocol, approximately 10^6^ mid-exponential phase *E. coli* and *Pseudoalteromonas* cells were washed twice with antibiotic-free mating medium (modified LB) and used for each conjugation. Chloramphenicol-resistant transconjugants were counted to calculate the transfer efficiency of pWD2-oriT. Colony PCR using pWD2-oriT-specific primers was used to confirm the presence of the transferred plasmid by isolating plasmids from randomly selected transconjugants. In addition, strain-specific RAPD-PCR was used to characterize and identify the resulting recipient strains to ensure no false positive colonies obtained. Conjugation was successful for all nine strains (Table 2, Additional file 1: Figure S2). At least twelve colonies from each recipient strain were randomly screened, and no false positive colonies were detected. Transfer efficiencies varied from 10^−6^ to 10^−3^ transconjugants per recipient cells (Table 2). Collectively, these results suggest that the mobilizable shuttle vector pWD2-oriT can be transferred to various *Pseudoalteromonas* strains by conjugation.

**Table 2:** **Transfer efficiencies of pWD2-oriT between** ***E. coli*** **and** ***Pseudoalteromonas*** **strains**

**Recipient strains**	CFU*	**Transconjugants** ^&^	**Efficiency**
A37-1-2	4.7 × 10^7^	3.0 × 10^4^	6.4 × 10^−4^
TAC125	2.5 × 10^7^	1.8 × 10^2^	7.2 × 10^−6^
SM20429	3.2 × 10^7^	1.4 × 10^4^	4.4 × 10^−4^
SM9913	3.8 × 10^7^	1.6 × 10^4^	4.2 × 10^−4^
DSM 16099	1.5 × 10^8^	1.6 × 10^3^	1.1 × 10^−5^
DSM 16098	9.5 × 10^7^	1.3 × 10^2^	1.4 × 10^−6^
DSM 6842	8.7 × 10^7^	9.4 × 10^1^	1.1 × 10^−6^
SCSIO 04301	9.7 × 10^7^	1.6 × 10^5^	1.6 × 10^−3^
SCSIO 11900	3.0 × 10^7^	9.8 × 10^1^	3.3 × 10^−6^

*Average number of recipient cells in two replicates.

^&^Average number of transconjugants in two replicate conjugations plated in triplicate plates; approximately 1/10^th^ of the total conjugation volume was plated.

### Construction of gene knockout vectors for *Pseudoalteromonas*

**Figure 2:**
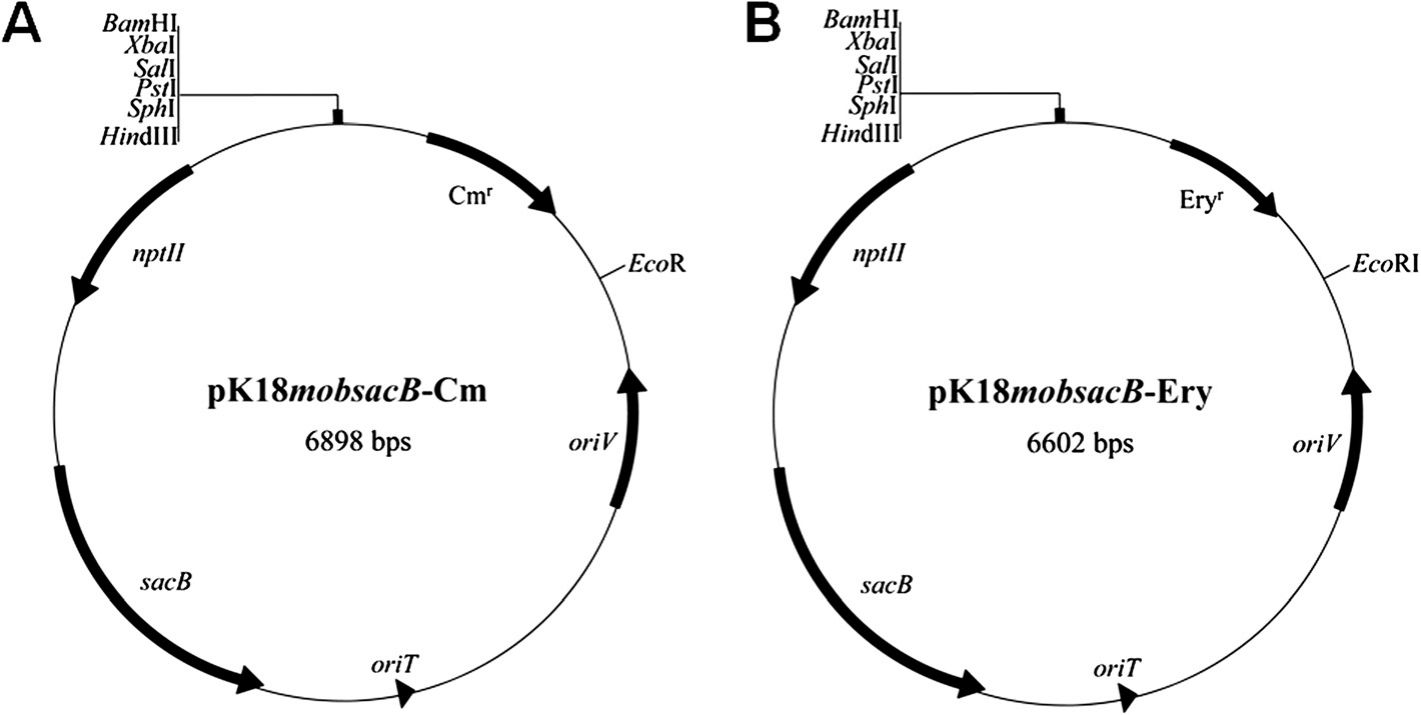
**Maps of the pk18** ***mobsacB*** **-Cm (A) and pk18** ***mobsacB*** **-Ery (B) suicide vectors.**

As shown in Figure 2, two derivatives vectors (pK18*mobsacB*-Cm and pK18*mobsacB*-Ery) were constructed by inserting chloramphenicol or erythromycin resistance genes into the *Bam*HI/*Eco*RI site of the gene knockout vector pK18*mobsacB* [24]. The vectors both contain the broad-host-range transfer machinery of plasmid RP4 and a modified *sacB* gene from *Bacillus subtilis* [24]. Gene *sacB* encodes levansucrase, which catalyzes the hydrolysis of sucrose and synthesizes levans. Levans are high molecular weight fructose polymers that are fatal to most Gram-negative bacteria [25]; *sacB* can therefore be used as a counter-selectable marker in these Gram-negative bacteria. The modified pK18*mobsacB* vector is suicidal in *Pseudoalteromonas* as its host range is restricted to *E. coli* and closely related species such as *Salmonella* and *Serratia*; therefore it can be used for gene knockout in *Pseudoalteromonas*. To verify the feasibility of this system, four *Pseudoalteromonas* strains, DSM 6842, SM9913, SCSIO 04301 and SCSIO 11900, covering high or low conjugation efficiencies for pWD2-oriT transfer, were used as recipient strains. To facilitate the verification of deletion mutants, we specifically selected gene or operon that may lead to phenotypic changes once disrupted as targets for functional confirmation.

### In-frame deletion of prodigiosin biosynthesis genes in DSM 6842

**Figure 3:**
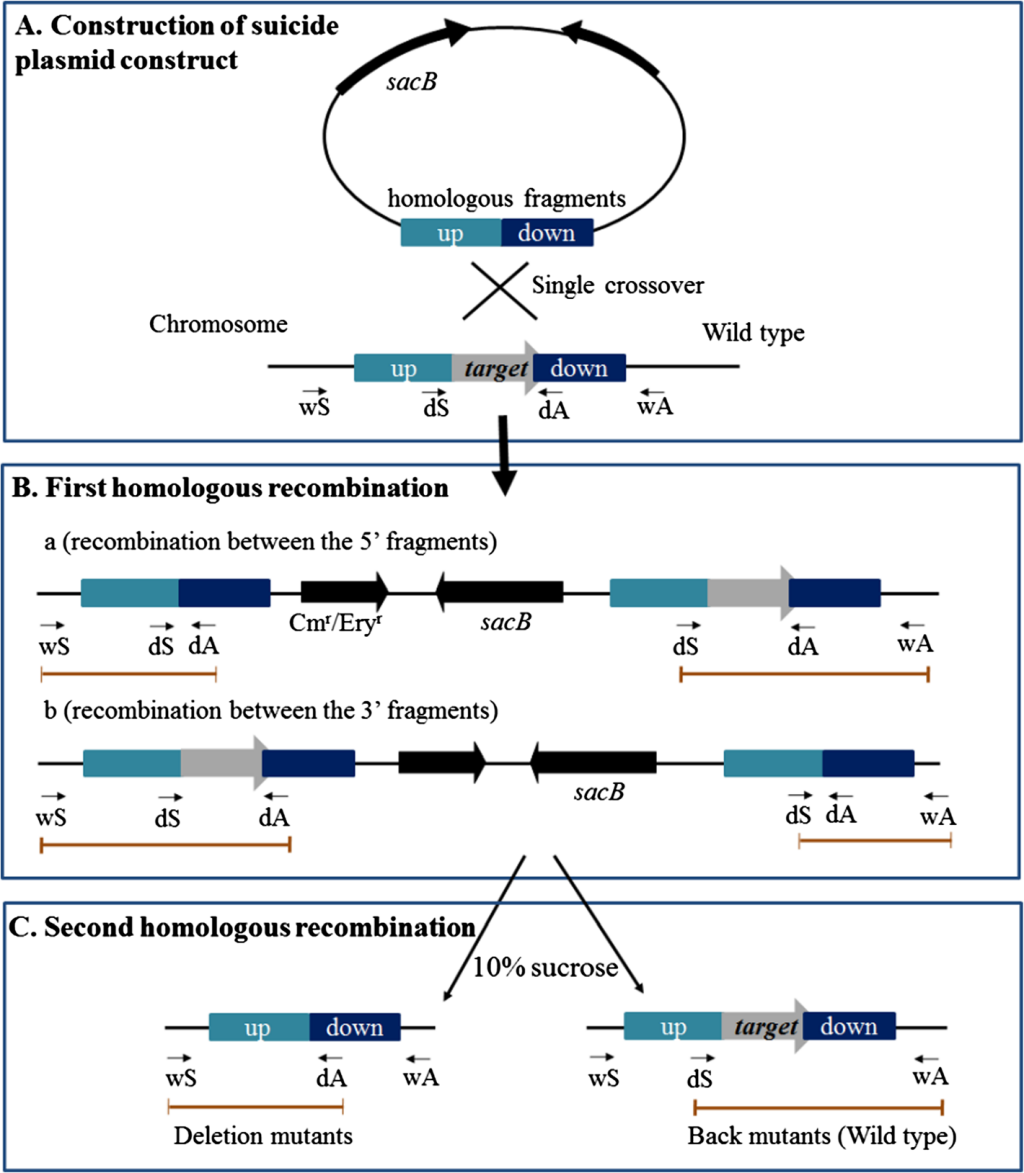
**Schematics illustrating the use of the pK18** ***mobsacB*** **-Cm or pK18** ***mobsacB*** **-Ery suicide plasmids to generate a defined, marker-free deletion in** ***Pseudoalteromonas*** **strains. (A)** Construction of suicide plasmid constructs containing the homologous fragments flanking the target DNA region. **(B)** First recombination event by integration into the chromosome via homologous recombination and position of the primers for plasmid insertion verification. **(C)** The second recombination event by sucrose selection, and position of primers to separate the deletion mutants from the wild-type.

**Figure 4:**
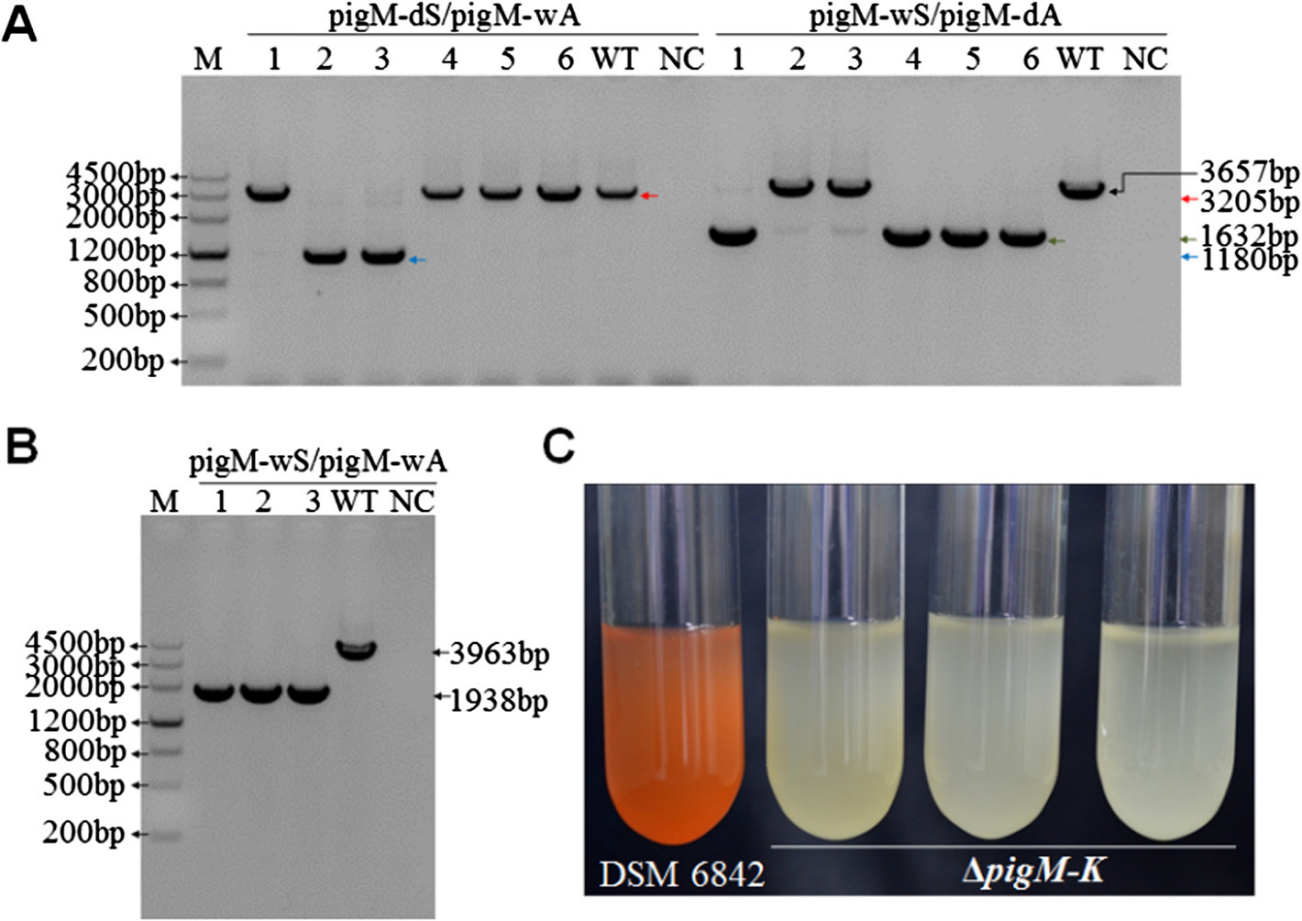
**Confirmation of in-frame deletion of prodigiosin biosynthesis genes in DSM 6842. (A)** PCR detection of *pigM-K* single crossover mutants using two primer pairs. M, DNA Marker III. 1–6, six independent colonies that can grow on the selective plate after conjugation. WT, DSM 6842 wild-type strain. NC, negative control ddH_2_O. **(B)** PCR confirmation of the mutants that underwent a second homologous recombination using the pigM-wS and pigM-wA primers. 1–3, three independent colonies after the second homologous recombination. WT, wild-type strain DSM 6842. NC, negative control ddH_2_O. **(C)** The prodigiosin production of three Δ*pigM-K* mutants and the wild-type strain DSM 6842.

To test whether this system can be used in DSM 6842, *pigM* and *pigK* genes in an operon related to prodigiosin biosynthesis [26] were selected as a target region for deletion. A schematic illustrating the use of pK18*mobsacB*-Ery or pK18*mobsacB*-Cm as the knockout vector is shown in Figure 3. The pK18*mobsacB*-Ery plasmid was used to construct a pK18Ery-*pigM-K* suicide plasmid containing the homologous fragments upstream and downstream of the target region, and was then mobilized into DSM 6842 by conjugation (Figure 3A). After mating, cells were plated on 2216E containing erythromycin to screen for clones in which a single crossover event occurred. Two different crossover events occurred simultaneously: colonies 1, 4, 5, and 6 were generated by 5′ recombination while colonies 2 and 3 were generated by 3′ recombination (Figure 4A and Figure 3B). Colony 1 from the first crossover was used for the second crossover. Sucrose counter-selection produced mutants without the *pigM*-*K* region (Figure 3C). As shown in Figure 4B, deletion in colonies 1–3 was confirmed by PCR using primer set pigM-wS/pigM-wA, followed by DNA sequencing to further validate complete removal of the target region from the host genome. The 2025 bp region containing *pigM*-*pigK* genes was deleted in the Δ*pigM-K* mutant. To check the phenotypic change of removing *pigM*-*K* region in DSM 6842, prodigiosin production was monitored in the Δ*pigM-K* strains and in the wild-type strain. As shown in Figure 4C, deletion mutants lost the ability to produce red pigment in 2216E medium. Thus conjugation-based genetic manipulation system is feasible for gene deletion in DSM 6842.

### In-frame deletion of *bsmA* in SM9913

**Figure 5:**
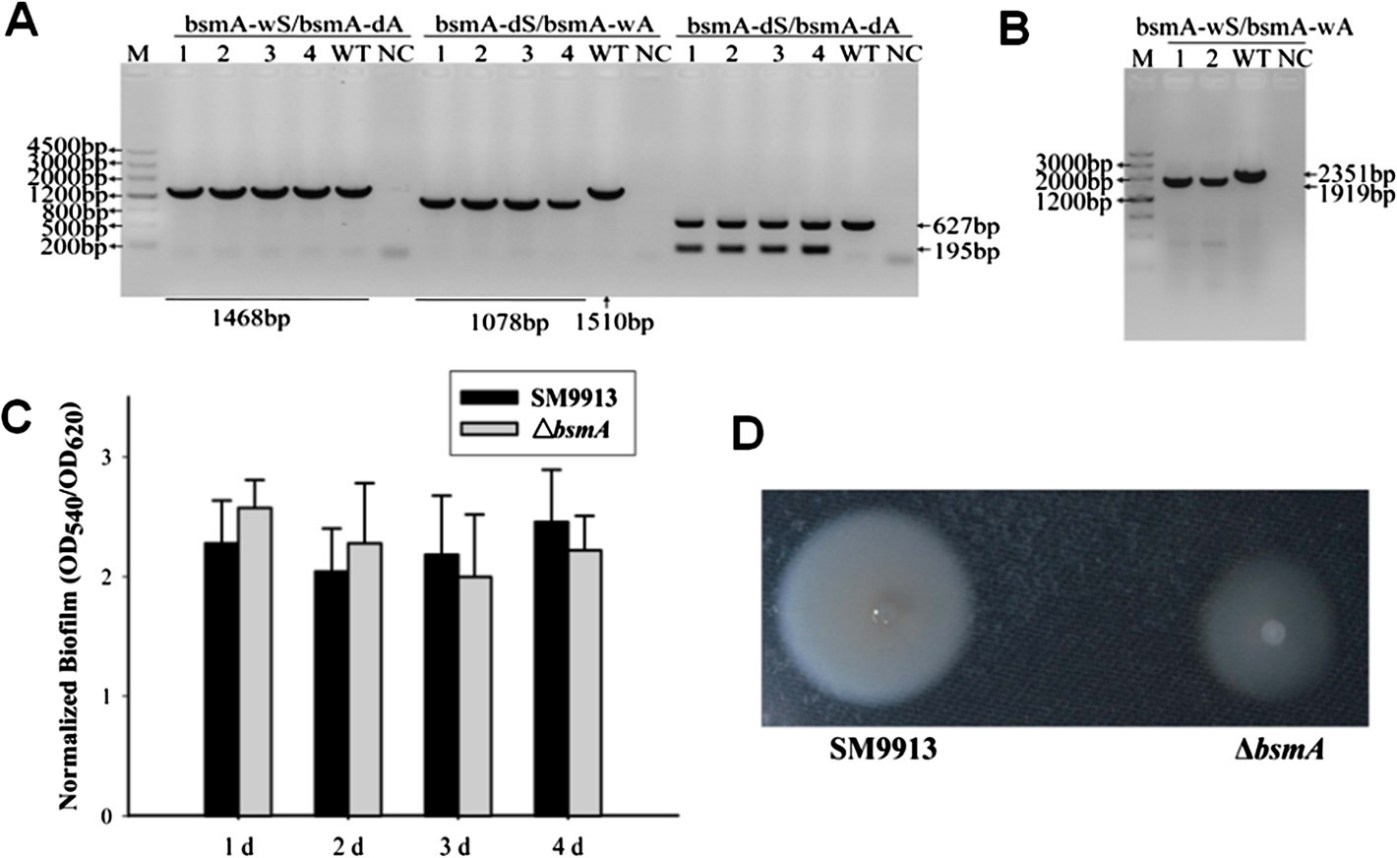
**Confirmation of in-frame deletion of** ***bsmA*** **in SM9913. (A)** PCR detection of *bsmA* single crossover mutants using three primer pairs. M, DNA Marker III. PCR templates used are: 1–4, four independent colonies that can grow on the selective plate after mating. WT indicates SM9913 wild-type strain. NC indicates negative control ddH_2_O. **(B)** PCR confirmation of the mutants that underwent a second homologous recombination using the bsmA-wS and bsmA-wA primers. 1–2, two independent colonies after the second homologous recombination. WT indicates SM9913 wild type. NC indicates negative control ddH_2_O. **(C)** Biofilm formation of wild type SM9913 and Δ*bsmA* strains*.* Normalized biofilm formation (total biofilm/growth) in SWLB medium at 20°C after 1 d, 2 d, 3 d, and 4 d in 96-well plates. Data are the average of ten replicate wells from two independent cultures, and one standard deviation is shown. **(D)** Swimming motility of wild type SM9913 and Δ*bsmA*.

To test the feasibility of gene knockout in SM9913, *bsmA*, which encodes *E. coli* homolog **b**iofilm **s**tress and **m**otility protein [27], was selected as a target gene. The pK18*mobsacB*-Cm plasmid was used to construct a pK18Cm-*bsmA* suicide plasmid containing the upstream and downstream regions of *bsmA.* Then pK18Cm-*bsmA* was transformed into *E. coli* WM3064, and mobilized into SM9913 by conjugation. Three sets of primers were used to confirm plasmid integration into the host genome. As shown in Figure 5A, pK18Cm-*bsmA* was integrated at the 3′ end in colonies 1–4 to generate a 1078 bp PCR product using primer set bsmA-dS/bsmA-wA, while the product length was 1510 bp for the wild-type SM9913. In addition, colonies 1–4 generated an extra 195 bp product using primer set bsmA-dS/bsmA-dA. Mutants from second homologous recombination were confirmed by PCR using primer set bsmA-wS/bsmA-wA. As shown in Figure 5B, after the removal of *bsmA*, PCR of Δ*bsmA* generated a 1919 bp product while the wild-type generated a 2351 bp product. To determine the effect of deleting *bsmA* on SM9913, biofilm formation was measured in the Δ*bsmA* strain. In contrast to *bsmA* in *E. coli* [27], deletion of *bsmA* in SM9913 did not significantly alter biofilm formation under similar test conditions (Figure 5C). However, Δ*bsmA* formed a smaller swimming halo when compared to the wild-type strain, indicating that BsmA increases swimming motility in SM9913 (Figure 5D).

### In-frame deletion of *hmgA* in SCSIO 04301

**Figure 6:**
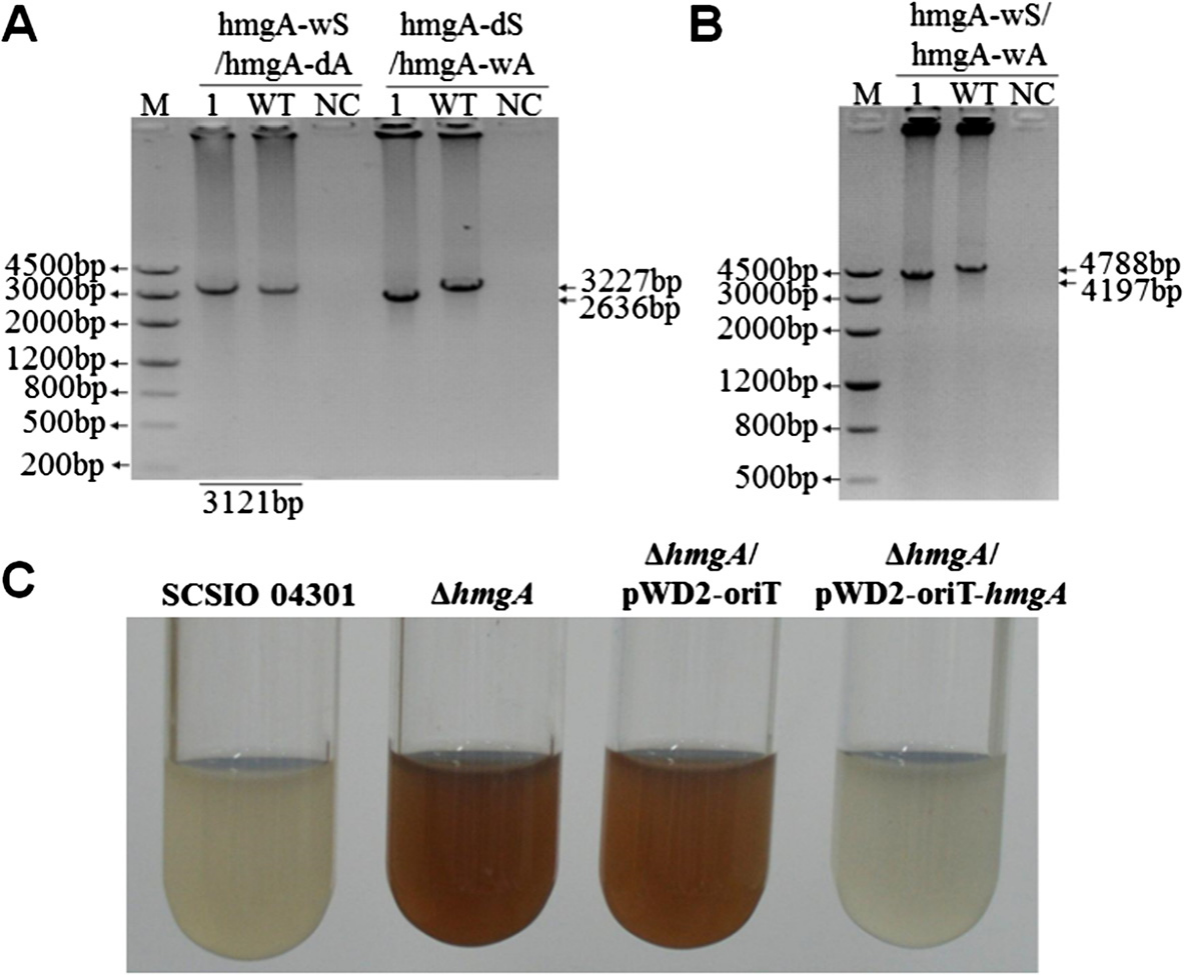
**Confirmation of in-frame deletion of** ***hmgA*** **in SCSIO 04301. (A)** PCR detection of *hmgA* single crossover mutants using two primer pairs. M, DNA Marker III. PCR templates used are: 1, one colony that can grow on the selective plate after mating. WT indicates SCSIO 04301 wild-type strain. NC indicates negative control ddH_2_O. **(B)** PCR confirmation of the mutants that underwent a second homologous recombination using the hmgA-wS and hmgA-wA primers. 1, one colony after the second homologous recombination. WT indicates SCSIO 04301 wild type. NC indicates negative control ddH_2_O. **(C)** The pigment production of wild-type SCSIO 04301, Δ*hmgA* mutant, Δ*hmgA/*pWD2-oriT and Δ*hmgA/*pWD2-oriT-*hmgA*.

To test the feasibility of gene knockout in SCSIO 04301, *hmgA*, encoding homogentisate 1, 2-dioxygenase [28], was selected as a target gene. As shown in Figure 6A, pK18Ery-*hmgA* was integrated at the 3′ end in colony 1 to generate a 2636 bp PCR product using the hmgA-dS/hmgA-wA primer set, while the product length was 3227 bp for the wild-type strain. As shown in Figure 6B, deletion in colony 1 was confirmed by PCR using the hmgA-wS/hmgA-wA primer set, followed by DNA sequencing to further validate complete removal of the target region from the host genome. The 591 bp internal *hmgA* region was deleted in the Δ*hmgA* mutant. To further confirm *hmgA* gene deletion in SCSIO 04301, pigment production was checked in the Δ*hmgA* mutant strain. As shown in Figure 6C, as expected, inactivation of *hmgA* leads to pyomelanin hyperproduction when cultured in 2216E medium for 48 h. In addition, complementation of *hmgA* via pWD2-oriT-*hmgA* to Δ*hmgA* mutant strain rescued the wild-type phenotype, while the empty vector pWD2-oriT failed to do that (Figure 6C). Thus gene deletion and gene complementation is feasible in SCSIO 04301.

### In-frame deletion of flagellar motor protein genes in SCSIO 11900

**Figure 7:**
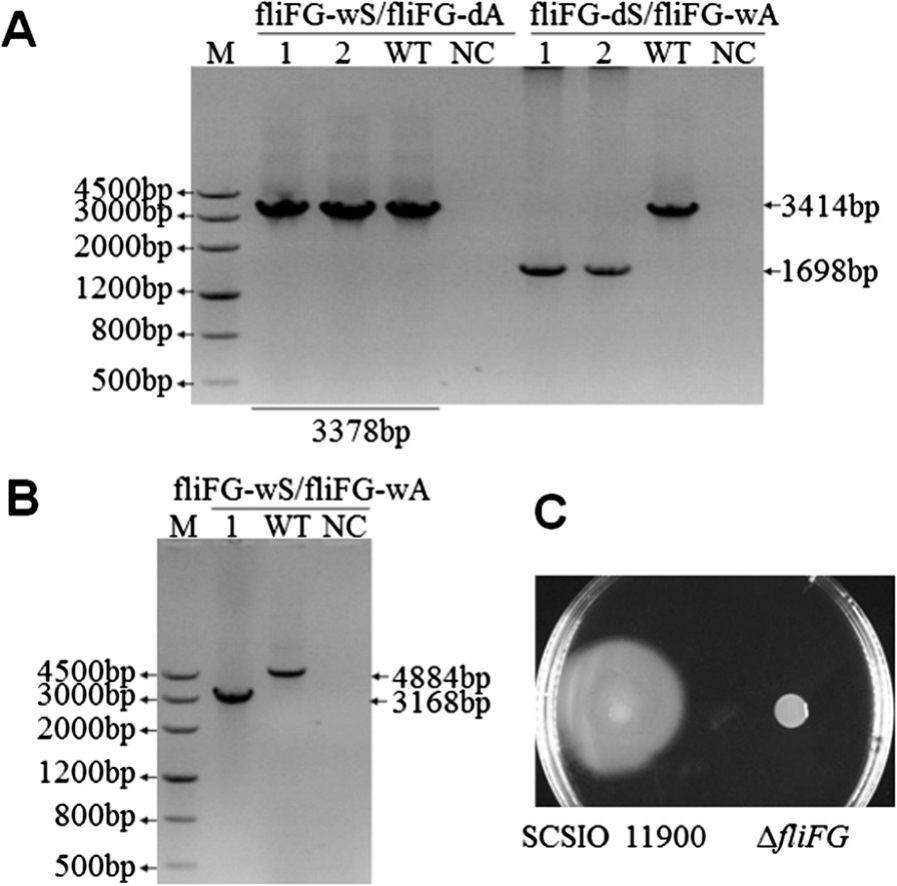
**Confirmation of in-frame deletion of flagellar motor protein genes in SCSIO 11900. (A)** PCR detection of *fliFG* single crossover mutants using two primer pairs. M, DNA Marker III. PCR templates used are: 1 and 2, two independent colonies that can grow on the selective plate after mating. WT indicates wild-type SCSIO 11900. NC indicates negative control ddH_2_O. **(B)** PCR confirmation of the mutants that underwent a second homologous recombination using the fliFG-wS and fliFG-wA primers. 1, one colony after the second homologous recombination. WT indicates wild-type SCSIO 11900. NC indicates negative control ddH_2_O. **(C)** Swimming motility of wild-type SCSIO 11900 and Δ*fliFG*.

To test whether this system can be used in SCSIO 11900, *fliF* and *fliG* genes in an operon encoding flagellar motor proteins [28] were selected as a target region for deletion. As shown in Figure 7A, pK18Ery-*fliFG* was integrated at the 3′ end in colony 1 to generate a 1698 bp PCR product using the fliFG-dS/fliFG-wA primer set, while the product length was 3414 bp for the wild-type strain. As shown in Figure 7B, deletion in colony 1 was confirmed by PCR using the primer set fliFG-wS/fliFG-wA. The 1716 bp region containing *fliFG* genes was deleted in the Δ*fliFG* mutant. To further confirm *fliFG* gene deletion in SCSIO 11900, motility was measured in the Δ*fliFG* mutant strain. As shown in Figure 7C, Δ*fliFG* mutant strain completely lost the ability of swimming motility compared with the wild-type strain. Thus conjugation-based genetic manipulation system is feasible for gene deletion in SCSIO 11900.

Different strain-specific genetic manipulation systems have been developed for *Pseudoalteromonas* strains TAC125 and SM9913 [14,15,29]. Here, we developed an efficient and generalizable genetic manipulation system for different *Pseudoalteromonas* strains. Incubation at 4°C has previously been used to select transconjugants and avoid overgrowth of the donor cells in the method developed for TAC125 [14,29]. However, conjugation at 4°C is unfeasible for knockout of genes essential for cold tolerance or adaptation [15], and many *Pseudoalteromonas* strains grow too slowly at 4°C. The mating temperature used in our approach was determined by the optimal growth temperature of the recipient strains, ranged from 20°C to 30°C, overcoming the limitations of cold temperature conditions. *E. coli* ET12567 (pUZ8002) containing a suicide plasmid was used as the donor strain for gene deletion in SM9913, however, control experiments showed that the donor cells alone can still grow on the selection plate due to lack of donor cell-specific selection pressure [15]. In this study, the auxotrophic *E. coli* WM3064 strain was used as donor which cannot grow without the addition of DAP, thus can eliminate interference from donor cells and reduce false positive rates.

Most *Pseudoalteromonas* strains are sensitive to chloramphenicol and erythromycin, including all the strains tested in this study. When used chloramphenicol in the selection of mutants from the the single crossover event, a few false positive colonies may appear, however, this can be easily avoided by using erythromycin for the same purpose. The precise mechanism remains unclear, but it might be attributed to differences in antibiotic killing mechanisms. Therefore, erythromycin selective marker vector is recommended over chloramphenicol for *Pseudoalteromonas* genetic manipulations.

## Conclusions

Here, we developed an efficient and generalizable genetic manipulation system for *Pseudoalteromonas* strains*.* Nine *Pseudoalteromonas* strains were sensitive to chloramphenicol and erythromycin, and thus both resistance genes were used to construct gene expression and gene knockout vectors. A conjugation transfer system was developed by modifying the culture conditions, which resulted in successful transfer of the pWD2-oriT gene expression vector from *E. coli* into *Pseudoalteromonas* strains with relatively high efficiency. By modifying a widely-used suicide plasmid (pK18*mobsacB*), a gene knockout system was developed and knockout of target DNA regions was confirmed in DSM 6842, SM9913, SCSIO 04301 and SCSIO 11900, covering from high or low conjugation efficiencies for pWD2-oriT transfer. In addition, gene complementation was also confirmed using this system. Taken together, conjugation-based genetic manipulation can be used efficiently for gene expression and gene deletion in *Pseudoalteromonas* strains, which will facilitate future *in vivo* studies of *Pseudoalteromonas*.

## Materials and methods

### Bacterial strains, plasmids, and growth conditions

The *Pseudoalteromonas* and *E. coli* strains and the plasmids used in this study are listed in Table 1. *E. coli* were cultured at 37°C in Luria-Bertani medium (LB) unless specified, and experiments with *Pseudoalteromonas* were conducted at 20-30°C in 2216E medium (BD Difco) or in SWLB medium (10 g peptone, and 5 g yeast extract dissolved in 1 l artificial seawater). DAP (diaminopimelic acid) was added at 0.3 mM to culture *E. coli* WM3064 strain. Conjugation assays were performed in modified LB mating medium (10 g peptone, 5 g yeast extract, 500 ml artificial seawater, and 500 ml distilled water) with 0.3 mM DAP. When needed, antibiotics were added at the following concentrations: 100 μg/ml for ampicillin (Amp), apramycin (Apr) and spectinomycin (Spc); 50 μg/ml for kanamycin (Kan); 30 μg/ml for chloramphenicol (Cm); 25 μg/ml for erythromycin (Ery); and 10 μg/ml for gentamycin (Gm) and tetracycline (Tet).

### Antibiotic sensitivity assay

Sensitivities of *Pseudoalteromonas* strains to eight antibiotics were tested (Additional file 1: Table S1). Strains were grown in 2216E medium at 20-30°C to late exponential phase and then diluted to 10^−2^-10^−7^ using 10-fold serial dilutions prior to plating on 2216E containing each antibiotic. The plates were incubated at 20-30°C for 48 h. Assays were performed in triplicate, and plates with no antibiotic were used as controls.

### Construction of pWD2-oriT and pWD2Ery-oriT mobilizable shuttle vectors

The mobilization module containing a *mob* gene and the corresponding *oriT* region was amplified from pBBR1MCS-2 using the oriT-F/oriT-R primer pair (Additional file 1: Table S2). The 1.5 kb PCR product was digested with *Bam*HI and inserted into the corresponding site of pWD2 [9], resulting in the mobilizable shuttle vector pWD2-oriT. To generate the pWD2Ery shuttle vector, the Ery resistance gene was amplified from pHT304 [30] using the Ery-F/Ery-R primer pair (Additional file 1: Table S2), and the 914 bp PCR product was digested with *Bam*HI/*Eco*RI and inserted into the corresponding sites of pWD2 [9]. To generate the pWD2Ery-oriT mobilizable shuttle vector, the 1.5 kb DNA region containing the mobilization module was derived from pWD2-oriT by digesting with *Bam*HI and inserted into the corresponding site of pWD2Ery.

### Conjugation assays

Conjugation experiments were performed as previously described [31] with some modifications. In brief, donor and recipient strains were grown to an OD_600_ of 0.8-1, and 2 ml donor cells and 1 ml recipient cells were harvested by centrifugation (4000 rpm for 3 min). Cells were washed twice with mating medium (MLB) and re-suspended in 100 μl MLB containing DAP. The donor and recipient cells were mixed briefly, the mixture dropped on MLB with DAP plates, and the plates were incubated for 8 h or more until a lawn was formed at 20-30°C. Cells were collected from the lawn and re-suspended in 2 ml 2216E medium and spread onto 2216E plates with appropriate antibiotics to select the transconjugants. Transfer efficiency was calculated as the ratio of transconjugants per recipient cells for each condition.

### PCR and RAPD-PCR analysis

Genomic DNA for PCR and random amplified polymorphic DNA (RAPD) analyses was isolated using a TIANamp Bacterial DNA Kit (Tiangen, Beijing, China). PCR primers are listed in Additional file 1: Table S2. Primers pWD2-S and pWD2-A were designed to amplify the replication region for detecting the pWD2-oriT plasmid in donor strains and transconjugants. RAPD-PCR analysis was performed using the 272 random primer (5′-AGCGGGCCAA-3′) [32] to identify donor strains, recipient strains, and transconjugants based on amplification profiles. The RAPD-PCR reaction was: 95°C for 5 min followed by 40 cycles of 95°C for 45 s, 36°C for 1 min, and 72°C for 2 min; and 72°C for an additional 10 min. Routine DNA manipulations were carried out following standard methods [33].

### Construction of the pK18*mobsacB*-Cm and pK18*mobsacB*-Ery suicide vectors

The 1.2 kb DNA region containing the Cm resistance gene was recovered from pWD2 with *Bam*HI/*Eco*RI and cloned into the commonly used pK18*mobsacB* vector for gene knockout [24], resulting in the pK18*mobsacB*-Cm suicide vector. The Ery resistance gene was amplified from pHT304 [30] using the Ery-F/Ery-R primer pair, and the 914 bp PCR product was digested with *Bam*HI/*Eco*RI and cloned into pK18*mobsacB* to produce the pK18*mobsacB*-Ery suicide vector.

### Construction of the Δ*pigM-K* mutant strain in DSM 6842

The suicide plasmid used for deletion of a DNA region containing *pigM*-*K* genes was based on pK18*mobsacB*-Ery. The schematic is shown in Figure 3. Two primer pairs (pigM-up-S/pigM-up-A and pigM-down-S/pigM-down-A) were used to amplify the upstream and downstream DNA sequences of the target region from DSM 6842 genomic DNA. The 996 bp and 815 bp PCR fragments were digested with *Xba*I/*Eco*RI and *Eco*RI/*Hind*III, respectively, cloned into the *Xba*I/*Hind*IIII sites of pK18*mobsacB*-Ery, and were transformed into *E. coli* WM3064. Suicide plasmid pK18Ery-*pigM-K* was mobilized from *E. coli* WM3064 into DSM 6842 by intergeneric conjugation. After mating, cells were spread on 2216E plates containing erythromycin (25 μg/ml) to screen for clones in which the suicide vector pK18Ery-*pigM-K* had integrated into the DSM 6842 genome via a single crossover event. The mutants were then grown at 25°C with shaking in 2216E medium without any antibiotics for 8 h. To select mutants in which the second recombination had occurred, the culture was diluted and spread on 2216E medium containing 10% sucrose and grown at 25°C for 24–36 h. Single colonies were transferred onto 2216E and 2216E containing 25 μg/ml erythromycin plates simultaneously, and colonies sensitive to erythromycin (25 μg/ml) were collected and confirmed by PCR followed by DNA sequencing.

Three other knockout mutants, Δ*bsmA* in SM9913, Δ*hmgA* in SCSIO 04301 and Δ*fliFG* in SCSIO 11900, were constructed using the similar steps as Δ*pigM-K*, and detailed procedures can be found in the Additional file 1. The procedure for the complementation of Δ*hmgA* can also be found in the Additional file 1.

### Crystal violet biofilm and motility assays

Biofilm formation was assayed in 96-well polystyrene plates (Corning Costar, Cambridge, MA) in SWLB medium at 20°C after 1 d, 2 d, 3 d, and 4 d with crystal violet staining [34]. To account for growth effects, biofilm formation was normalized by dividing the total biofilm by the maximal bacterial growth as measured by turbidity at 620 nm for each strain. Cell motility was examined with 1% tryptone and 0.3% agar dissolved in seawater medium. Motility halos were quantified after 16 h using at least three plates for each condition and two independent cultures for each strain.

## Additional file

Additional file 1: Table S1. Antibiotic resistance of *Pseudoalteromonas* strains. **Table S2.** Primers used in this study. **Figure S1.** Growth test of the *E. coli* WM3064 donor strain and four recipient strains (DSM 16099, DSM 16098, SM9913, and DSM 6842) in LB with or without DAP, modified LB medium (MLB) with DAP, and 2216E with or without DAP. **Figure S2.** Confirmation of transconjugants by PCR. (A) PCR amplification with the pWD2-S/pWD2-A primer pair to detect pWD2-oriT in the transconjugants. (B) RAPD-PCR to confirm the host strain of the transconjugants. The templates in (A) and (B) are: Lane 1, donor strain *E. coli* WM3064/pWD2-oriT; Lane 2, SM9913; Lane 3-4, SM9913/pWD2-oriT; Lane 5, A37-1-2; Lane 6-7, A37-1-2/pWD2-oriT; Lane 8, DSM 16099; Lane 9-10, DSM 16099/pWD2-oriT; Lane 11, DSM 16098; Lane 12-13, DSM 16098/pWD2-oriT; Lane 14, SM20429; Lane 15-16, SM20429/pWD2-oriT; Lane 17, TAC125; Lane 18-19, TAC125/pWD2-oriT; Lane 20, DSM 6842; Lane 21-22, DSM 6842/pWD2-oriT. Lane 23, SCSIO 04301; Lane 24-25, SCSIO 04301/pWD2-oriT; Lane 26, SCSIO 11900; Lane 27-28, SCSIO 11900/pWD2-oriT; M1, DL2K marker; M2, DNA Marker III.
