# Supplementary material for: Development of an efficient conjugation-based genetic manipulation system for Pseudoalteromonas
Source: Microb Cell Fact. 2015 Jan 23;14:11. doi: 10.1186/s12934-015-0194-8 (PMC4318363; doi:10.1186/s12934-015-0194-8)
Supplement: Additional file 1: Table S1. — Antibiotic resistance of Pseudoalteromonas strains. Table S2. Primers used in this study. Figure S1. Growth test of the E. coli WM3064 donor strain and four recipient strains (DSM 16099, DSM 16098, SM9913, and DSM 6842) in LB with or without DAP, modified LB medium (MLB) with DAP, and 2216E with or without DAP. Figure S2. Confirmation of transconjugants by PCR. (A) PCR amplification with the pWD2-S/pWD2-A primer pair to detect pWD2-oriT in the transconjugants. (B) RAPD-PCR to confirm the host strain of the transconjugants. The templates in (A) and (B) are: Lane 1, donor strain E. coli WM3064/pWD2-oriT; Lane 2, SM9913; Lane 3-4, SM9913/pWD2-oriT; Lane 5, A37-1-2; Lane 6-7, A37-1-2/pWD2-oriT; Lane 8, DSM 16099; Lane 9-10, DSM 16099/pWD2-oriT; Lane 11, DSM 16098; Lane 12-13, DSM 16098/pWD2-oriT; Lane 14, SM20429; Lane 15-16, SM20429/pWD2-oriT; Lane 17, TAC125; Lane 18-19, TAC125/pWD2-oriT; Lane 20, DSM 6842; Lane 21-22, DSM 6842/pWD2-oriT. Lane 23, SCSIO 04301; Lane 24-25, SCSIO 04301/pWD2-oriT; Lane 26, SCSIO 11900; Lane 27-28, SCSIO 11900/pWD2-oriT; M1, DL2K marker; M2, DNA Marker III. [file 12934_2015_194_MOESM1_ESM.pdf]

Supplementary File

Development of an efficient conjugation-based genetic manipulation system for *Pseudoalteromonas*

Pengxia Wang[1], Zichao Yu[2], Baiyuan Li[1,3], Xingsheng Cai[1], Zhenshun Zeng[1,3], Xiulan Chen[2] and Xiaoxue Wang[1]*

[1] Key Laboratory of Tropical Marine Bio-resources and Ecology, the South China Sea Institute of Oceanology, Chinese Academy of Sciences, Guangzhou 510301, China

[2] State Key Laboratory of Microbial Technology, Marine Biotechnology Research Center, Shandong University, Jinan 250100, China

[3] University of Chinese Academy of Sciences, Beijing 100049, China

*To whom correspondence should be addressed. E-mail: xxwang@scsio.ac.cn

Table S1. Antibiotic resistance of *Pseudoalteromonas* strains.

Strain	Amp	Apr	Cm	Ery	Gm	Kan	Spc	Tet
A37-1-2	+	++	-	-	+++	+++	+++	+++
DSM 16099	+	+	-	-	++	++	+	+++
DSM 16098	+	++	-	-	+++	+++	+++	+++
DSM 6842	-	+++	-	-	+++	+++	++	+++
SM20429	-	+	-	-	+++	+++	+++	+++
SM9913	+	++	-	-	+++	+++	+++	+++
TAC125	-	+	-	-	++	++	+++	+++
SCSIO 04301	+++	+++	-	-	+	++	+	+++
SCSIO 11900	++	+++	-	-	++	++	+	+++

Amp (ampicillin, 100 μg/ml); Apr (apramycin, 100 μg/ml); Cm (chloramphenicol, 30 μg/ml); Ery (erythromycin, 25 μg/ml); Gm (gentamycin, 10 μg/ml); Kan (kanamycin, 50 μg/ml); Spc (spectinomycin, 100 μg/ml); Tet (tetracycline, 10 μg/ml).

(-, no growth; +, growth)

Table S2. Primers used in this study.

Primers	Sequence (5'-3')	Purpose	Expected size (bp)
oriT-F	CGGGATCCGGGTATGTGGTCGAAGGCTG	Construction of pWD2-oriT	1506
oriT-R	CGGGATCCCAGCTGGCAATTCCGGTTCG		
pWD2-S	GAATTTCAACGGCATTCAAGC	Detection of pWD2-oriT	428
pWD2-A	GACCTTTTAGATCCAGCTCC		
Ery-F	CGGGATCCCCAGTGACTAATCTTATGAC	Construction of pK18*mobsacB*-Ery and pWD2Ery-oriT	914
Ery-R	CGGAATTCCTCCATTCCCTTTAGTAACG		
bsmA-up-S	CCCAAGCTTCTTTCTGATGTGACAGGCGAG	Upstream homologous fragment of *bsmA*	753
bsmA-up-A	ACATGCATGCCTGGTTGTTTAATGCGGTC		
bsmA-down-S	ACATGCATGCGAAGGTGGTGAGCTATTGG	Downstream homologous fragment of *bsmA*	933
bsmA-down-A	CGCGTCGACGGTGTAAACCCTTCTAAGTG		
bsmA-wS	CCCAGAGCTAATGGAAGCTG	Confirmation of Δ*bsmA*	2351[a]
bsmA-wA	GCGTGAGTGACTGTCATCAAG		1919[b]
bsmA-dS	GCCACCGCATTAAACAACCAG	Confirmation of the pK18Cm-*bsmA* integration	627[a]
bsmA-dA	CCAGCAGTTAGTACAAAGCAATC		195[b]
pigM-up-S	GCTCTAGAGAATGCGGGTAAGTAAAGA	Upstream homologous fragment of *pigM-K* region	966
pigM-up-A	CGGAATTCAAACCATTGCGTAGTTGTAA		
pigM-down-S	CGGAATTCCGCCCAGGTGCTGAATGTG	Downstream homologous fragment of *pigM-K* region	815
pigM-down-A	CCCAAGCTTCAGCCTGTGCCAGTTCCAT		
pigM-wS	CAAGCCCTACAGCCAACAT	Confirmation of Δ*pigM-K*	3963[a]
pigM-wA	CGGAACATCTGAAGATCCCAC		1938[b]
pigM-dS	TGCTGACGCCTTTGTGCTG	Confirmation of the pK18Ery-*pigM-K* integration	2899[a]
pigM-dA	ATGCCATTTCGGGTTCCTG		874[b]
hmgA-up-S	GCTCTAGACTGAAATAGGGCTTACTGG	Upstream homologous fragment of *hmgA*	1954
hmgA-up-A	AACTGCAGTTGTGCATTTGCGCTACCATTT		
hmgA-down-S	AACTGCAGAACACTTTCCGCCCACCCTATT	Downstream homologous fragment of *hmgA*	2054
hmgA-down-A	CCCAAGCTTCAATGTCACAAACGGCTCAA		
hmgA-wS	AAGGTTTGGATTTTTCTTTTCG	Confirmation of Δ*hmgA*	4788[a]
hmgA-wA	GTTGAAGTCACCTACTCACCTC		4197[b]
hmgA-dS	TCGCTTTCATCTCTTTTGTC	Confirmation of the pK18Ery-*hmgA* integration	1560[a]
hmgA-dA	TAGGCATCGCTTTGCTGTTC		969[b]
fliFG-up-S	ACATGCATGCGCTAATAAAGTTGCTCGATC	Upstream homologous fragment of *fliFG* region	1512
fliFG-up-A	CGGAATTCAATAATTCTCCACTACCTAGTT		
fliFG-down-S	CGGAATTCTCAAGAACAAAAACAACTACC	Downstream homologous	1511

fliFG-down-A	ACGCGTCGACGTACCAGCTGTCTTTCTGCT	fragment of *fliFG* region	
fliFG-wS	GCTAAGCCATTTGCACCAG	Confirmation of Δ*fliFG*	4884[a]
fliFG-wA	GGCTATGATTGCTTCGCAA		3168[b]
fliFG-dS	GCCGCTTTATCTACACCAT	Confirmation of the	1908[a]
fliFG-dA	GCTTCACTGAGCATAGTGGCT	pK18Ery-*fliFG* integration	192[b]
hmgA-pWD2-oriT-F	ACATGCATGCTCGACCATATCGCTGCCATCACG	Complementation of Δ*hmgA*	1755
hmgA-pWD2-oriT-R	AACTGCAGTTATACGTCCTGTTTACCTGTA		

Restriction sites included in oligonucleotide sequences are underlined.

[a] the size of the wild-type.

[b] the size of the deletion mutant.

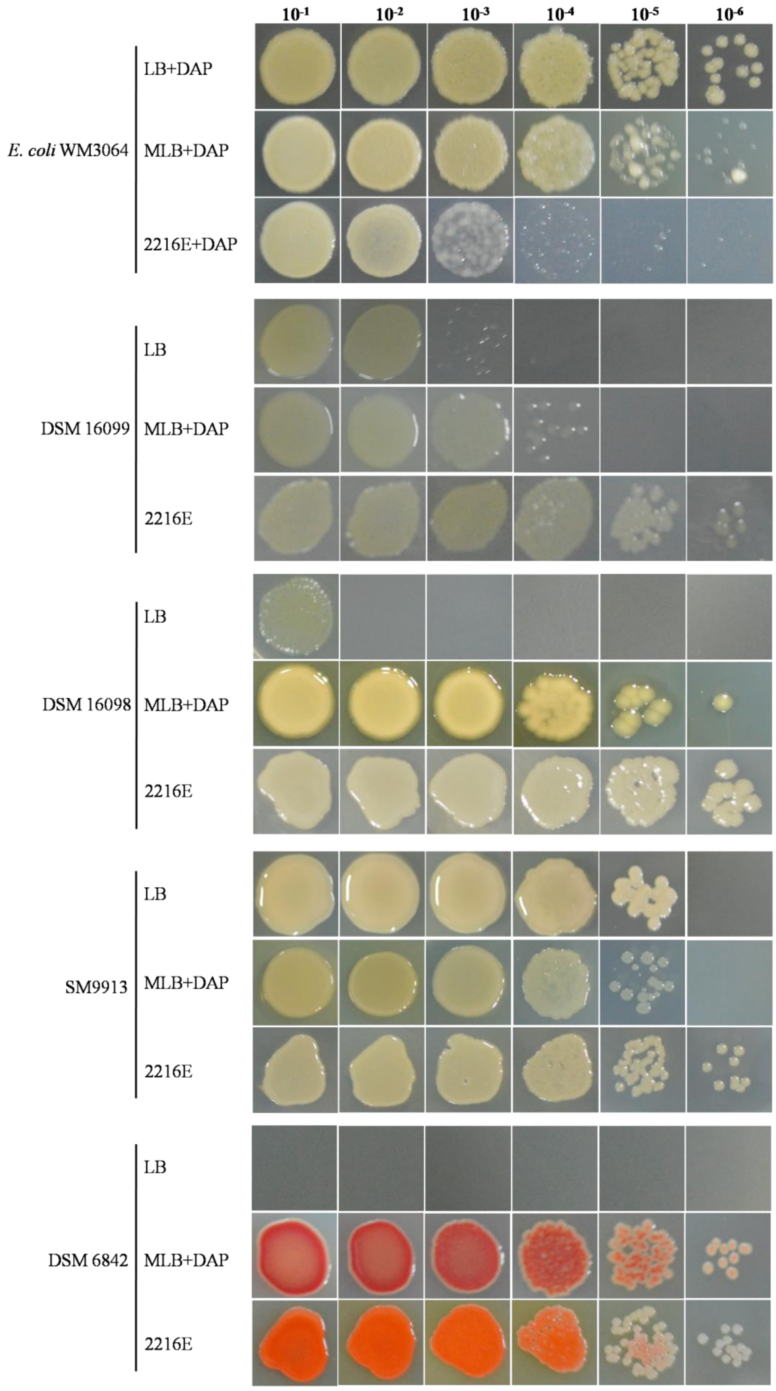

Fig. S1. Growth test of the *E. coli* WM3064 donor strain and four recipient strains (DSM 16099, DSM 16098, SM9913, and DSM 6842) in LB with or without DAP, modified LB medium (MLB) with DAP, and 2216E with or without DAP.

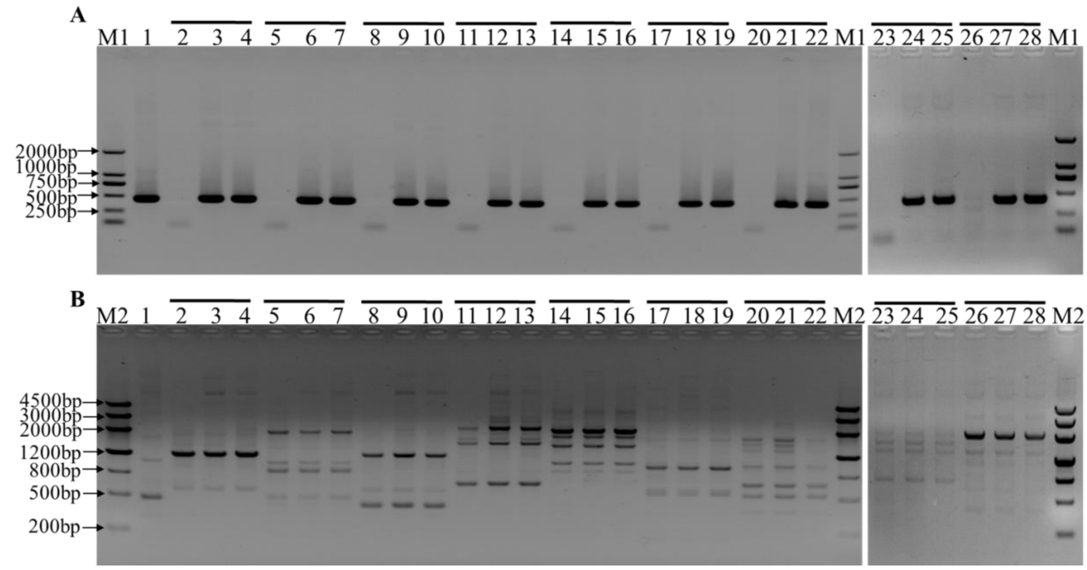

Fig. S2. Confirmation of transconjugation by PCR. (A) PCR amplification with the pWD2-S/pWD2-A primer pair to detect pWD2-oriT in the transconjugants. (B) RAPD-PCR to confirm the host strain of the transconjugants. The templates in (A) and (B) are: Lane 1, donor strain *E. coli* WM3064/pWD2-oriT; Lane 2, SM9913; Lane 3-4, SM9913/pWD2-oriT; Lane 5, A37-1-2; Lane 6-7, A37-1-2/pWD2-oriT; Lane 8, DSM 16099; Lane 9-10, DSM 16099/pWD2-oriT; Lane 11, DSM 16098; Lane 12-13, DSM 16098/pWD2-oriT; Lane 14, SM20429; Lane 15-16, SM20429/pWD2-oriT; Lane 17, TAC125; Lane 18-19, TAC125/pWD2-oriT; Lane 20, DSM 6842; Lane 21-22, DSM 6842/pWD2-oriT. Lane 23, SCSIO 04301; Lane 24-25, SCSIO 04301/pWD2-oriT; Lane 26, SCSIO 11900; Lane 27-28, SCSIO 11900/pWD2-oriT; M1, DL2K marker; M2, DNA Marker III.

Construction of the Δ*bsmA* strain in SM9913.

The suicide plasmid used for *bsmA* gene knockout was based on pK18*mobsacB*-Cm; the schematic is shown in **Fig. 3.** Two primer pairs (bsmA-up-S/bsmA-up-A and bsmA-down-S/bsmA-down-A) were used to amplify the upstream and downstream DNA sequences of *bsmA* from SM9913 genomic DNA. The 753 bp and 933 bp PCR fragments were digested with *Hin*dIII/*Sph*I and *Sph*I/*Sal*I, respectively, and cloned into the *Hin*dIII/*Sal*I sites of pK18*mobsacB*-Cm. These were transformed into *E.coli* WM3064 to yield the WM3064/pK18Cm-*bsmA* recombinant strain for *bsmA* gene knockout.

The pK18Cm-*bsmA* suicide vector was mobilized from *E. coli* WM3064 into SM9913 by intergeneric conjugation. After mating, the cells were spread on 2216E plates containing chloramphenicol (30 μg/ml) to screen for clones in which the suicide plasmid pK18Cm-*bsmA* had inserted into the SM9913 genome via a single crossover event. Mutants were then grown at 20°C with shaking in 2216E medium without antibiotics for 8 h. To select the mutants in which the second recombination had occurred, the culture was diluted and spread on 2216E containing 10% sucrose and grown at 20°C for about 36-48 h. Single colonies were then transferred onto 2216E and 2216E containing chloramphenicol (30 μg/ml) plates simultaneously, and the colonies sensitive to chloramphenicol were collected and confirmed by PCR followed by DNA sequencing.

Construction of the Δ*hmgA* strain in SCSIO 04301 and complementation of Δ*hmgA*. The suicide plasmid used for *hmgA* gene knockout was based on pK18*mobsacB*-Ery; the schematic is shown in **Fig. 3**. Two primer pairs (hmgA-up-S/hmgA-up-A and hmgA-down-S/hmgA-down-A) were used to amplify the upstream and downstream DNA sequences of *hmgA* from SCSIO 04301

genomic DNA. The 1954 bp and 2054 bp PCR fragments were digested with *Xba*I/*Pst*I and *Pst*I/*Hin*dIII, respectively, and cloned into the XbaI/*Hin*dIII sites of pK18*mobsacB*-Ery. These were transformed into *E.coli* WM3064 to yield the WM3064/pK18Ery-*hmgA* recombinant strain for *hmgA* gene knockout.

The pK18Ery-*hmgA* suicide vector was mobilized from *E. coli* WM3064 into SCSIO 04301 by intergeneric conjugation. After mating, the cells were spread on 2216E plates containing erythromycin (25 μg/ml) to screen for clones in which the suicide plasmid pK18Ery-*hmgA* had inserted into the SCSIO 04301 genome via a single crossover event. Mutants were then grown at 25°C with shaking in 2216E medium without antibiotics for 8 h. To select the mutants in which the second recombination had occurred, the culture was diluted and spread on 2216E containing 10% sucrose and grown at 25°C for about 36-48 h. Single colonies were then transferred onto 2216E and 2216E containing erythromycin (25 μg/ml) plates simultaneously, and the colonies sensitive to erythromycin were collected and confirmed by PCR using appropriate primers and sequencing.

For complementation of Δ*hmgA*, the *hmgA* gene with its promoter was amplified from SCSIO 04301 using the primers hmgA-pWD2-oriT-F and hmgA-pWD2-oriT-R. The 1755 bp PCR fragment was digested with *Sph*I and *Pst*I, and cloned into the corresponding sites of pWD2-oriT. These were transformed into *E.coli* WM3064 to yield the WM3064/pWD2-oriT-*hmgA* recombinant strain. The pWD2-oriT-*hmgA* complementation vector was mobilized from *E. coli* WM3064 into Δ*hmgA* by intergeneric conjugation. After mating, the cells were spread on 2216E plates containing chloramphenicol (30 μg/ml) to screen for clones in which the pWD2-oriT-*hmgA* had transferred into Δ*hmgA*. As a control, the empty vector pWD2-oriT was transferred into the Δ*hmgA* as the similar

steps. Colony PCR using pWD2-oriT-specific primers was used to confirm the presence of the transferred plasmid.

Construction of the Δ*fliFG* mutant strain in SCSIO 11900. The suicide plasmid used for deletion of a DNA region containing *fliF and fliG* genes was based on pK18*mobsacB*-Ery. The schematic is shown in **Fig. 3**. Two primer pairs (fliFG-up-S/fliFG-up-A and fliFG-down-S/fliFG-down-A) were used to amplify the upstream and downstream DNA sequences of the target region in SCSIO 11900 genomic DNA. The 1512 bp and 1511 PCR fragments were digested with *Sph*I/*Eco*RI and *Eco*RI/*Sal*I, respectively, and cloned into the *Sph*I/*Sal*I sites of pK18*mobsacB*-Ery. These were transformed into *E. coli* WM3064 to yield the WM3064/pK18Ery-*fliFG* recombinant strain.

The suicide plasmid pK18Ery-*fliFG* was mobilized from *E. coli* WM3064 into SCSIO 11900 by intergeneric conjugation. After mating, cells were spread on 2216E plates containing erythromycin (25 μg/ml) to screen for clones in which the suicide vector pK18Ery-*fliFG* had inserted into the SCSIO 11900 genome via a single crossover event. The mutants were then grown at 25°C with shaking in 2216E medium without antibiotics for 8 h. To select mutants in which the second recombination had occurred, the culture was diluted and spread on 2216E medium containing 10% sucrose and grown at 25°C for about 24-36 h. Single colonies were transferred onto 2216E and 2216E containing erythromycin (25 μg/ml) plates simultaneously, and colonies sensitive to erythromycin (25 μg/ml) were collected and confirmed by PCR followed by DNA sequencing.
